# Clinical Outcomes and Characteristics of COVID-19 in Neonates: A Single-Center Study in Romania

**DOI:** 10.3390/life14121650

**Published:** 2024-12-12

**Authors:** Maria Elena Cocuz, Iuliu-Gabriel Cocuz, Ligia Rodina, Ruxandra Filip, Florin Filip

**Affiliations:** 1 Fundamental Prophylactic and Clinical Disciplines Department, Faculty of Medicine, Transilvania University of Brasov, 500003 Brasov, Romania; maria_elenacocuz@yahoo.com; 2 Clinical Pneumology and Infectious Diseases Hospital of Brasov, 500118 Brasov, Romania; ligiarodina@yahoo.com; 3 Pathophysiology Department, “George Emil Palade” University of Medicine, Pharmacy, Sciences and Technology of Targu Mures, 540142 Targu Mures, Romania; 4 Pathology Department, Mures Clinical County Hospital, 540011 Targu Mures, Romania; 5 Faculty of Medicine, “George Emil Palade” University of Medicine, Pharmacy, Sciences and Technology of Targu Mures, 540142 Targu Mures, Romania; ruxandra.filip@aol.com; 6 Emergency Hospital‚ Sf. Ioan Cel Nou’, 720224 Suceava, Romania; florin.filip@usm.ro; 7 Faculty of Medicine and Biological Sciences, Stefan Cel Mare University of Suceava, 720229 Suceava, Romania

**Keywords:** SARS-CoV-2 infection, COVID-19, neonates

## Abstract

Background: SARS-CoV-2 infection is generally associated with less severe forms of disease in children, where most cases only require symptomatic treatment. However, there is a paucity of information regarding the impact and clinical course of COVID-19 in neonate patients. This study aimed to analyze the epidemiological and clinical aspects of COVID-19 in this particular age group who were patients treated in our department. Materials and methods: This is a retrospective observational study that includes neonates (aged less than 1 month) who were diagnosed with COVID-19. The patients were admitted between 1 January 2022 and 31 December 2023, to the Infectious Diseases Pediatric Department of the Hospital Clinic of Pneumophthisiology and Infectious Diseases in Brașov, Romania. All the patients were tested for SARS-CoV-2 infection at admission, using either a real-time PCR (RT-PCR) or rapid antigen testing, according to the national COVID-19 protocol in use at the time. We collected the following data: demographic data, clinical picture and laboratory values at presentation, clinical course, complications, and other significant data. All the data were extracted from existing hospital administrative databases or electronic medical records. Results: Nine neonates were hospitalized with COVID-19, of which five were boys, and four were girls; the mean age was 18.89 days (ranging between 6 and 28 days). The clinical picture at admission mainly consisted of fever (eight cases) and nasal obstruction and cough (five cases each). Only one patient required oxygen support. Co-infections with *Streptococcus pneumoniae* and *Haemophilus influenzae* (one case), respiratory syncytial virus (RSV, one case), and rotavirus (one case) were identified. Complications were represented by acute bronchiolitis in three patients. Biologically, lymphopenia was found in three cases, monocytosis in five cases, and increased ferritin values in five cases. The clinical outcome was favorable in all the cases. The patients were discharged in improved condition after an average stay of 5.11 days (ranging between 3 and 10 days). Conclusions: Our data support the observation that infection with SARS-CoV-2 in neonates is a relatively benign condition with a good prognosis. Our study has several limitations and establishes a foundation for future studies on a larger sample of term and premature neonates with different comorbidities.

## 1. Introduction

The coronavirus disease 2019 (COVID-19) pandemic seriously challenged healthcare systems worldwide [1,2]. Children and adolescents experienced less severe forms of the disease than adults, and most cases only required symptomatic treatment or no treatment [1,2,3,4]. The mortality rates in children were related to certain risk factors and were low [5,6,7]. However, there are few reports on SARS-CoV-2 infection in young children, especially in neonates. Most articles on this subject include a small number of cases or present isolated experiences from single institutions. This article presents our experience with SARS-CoV-2 infection in nine children aged less than 1 month, and analyzes the epidemiological and clinical aspects of COVID-19 in this age group. We consider an important aspect, namely, the lack of an approved etiological therapy for the treatment [8,9] of COVID-19 in newborns at the time of the hospitalization of the patients included in this study. Thus, our study presents an analysis of the natural evolution of SARS-CoV-2 infection in the absence of a specific etiological treatment, possibly only influenced by the pathogenetic therapies targeting the co-infections, providing information to better monitor and treat COVID-19 in newborns.

## 2. Materials and Methods

We conducted a retrospective study on newborns who were hospitalized with COVID-19 in the Pediatric Infectious Diseases ward of the Clinical Hospital for Pneumophtysiology and Infectious Diseases in Brașov, Romania, between 1 January 2022 and 31 December 2023. The diagnosis of COVID-19 was established on admission to the hospital in all the patients by means of a rapid antigen test and/or RT-PCR, according to the national protocols for COVID-19 in place at the time. Informed consent for admission, treatment, and the use of data for scientific research was obtained at admission from the legal guardians of the patients. The approval of the Ethics Commission of the hospital was obtained for this study (No. 1871, 7 February 2024). The patients were de-identified. The data used in this study were extracted from the patients’ clinical observation sheets and electronic medical records. The collected data were the age (at the time of the clinical onset of the disease, at the time of the diagnosis of COVID-19, and at the time of admission to the hospital), sex, personal pathological history, type of birth (natural or by cesarean section, at term or premature), co-infection with SARS-CoV-2 in the mother, clinical manifestations, clinical form of the disease, co-infections, hospitalization in the pediatric ward or intensive care unit (PICU), the need for additional oxygen (O_2_), and the length of stay (LoS). We analyzed the values of selected parameters determined by laboratory analyses at admission (serum leukocyte count, C-reactive protein, alanine aminotransferase/glutamate–pyruvate transaminase (ALT/GPT), lactate dehydrogenase (LDH), ferritin, serum creatinine, and thrombocyte count), as well as any changes in the imaging investigation results, namely, chest X-ray images. All the laboratory values presented in our study were interpreted from a qualitative point of view, defining them as normal, increased, or decreased in comparison to average values, due to the fact that many of the laboratory tests were performed by different laboratories (Clinical Hospital for Pneumophthysiology and Infectious Diseases in Brașov and the Laboratory of the Emergency Clinical Hospital for Children in Brasov) with different ranges of normal values, according to each kit that was used.

The severity of the clinical form of COVID-19 was evaluated according to the existing national protocols for the diagnosis and treatment of COVID-19 at the time of the hospitalization of the patients [8,9]:

- Mild form: general and/or upper respiratory tract symptoms, without manifestations that are evocative of pneumonia and without lung damage;
- Medium form: patients with pneumonia confirmed by imaging but without hypoxemia (if there was no respiratory impairment prior to the current illness);
- Severe form: respiratory distress with arterial oxygen saturation (SaO_2_) below 94% in atmospheric air and imaging abnormalities indicative of lung damage;
- Critical form: patients with severe respiratory failure requiring ventilatory support, septic shock, and/or multiple organ dysfunction.

## 3. Results

Between 1 January 2022 and 31 December 2023, 278 children with COVID-19 were admitted to the Pediatric Infectious Diseases Department, out of which 9 (3.23%) were newborns (2 were admitted in 2022, and 7 in 2023). The diagnosis of SARS-CoV-2 infection was established at hospital admission using a rapid antigen test for six children, an RT-PCR for two children, and both types of tests for one child.

The time interval between the clinical onset of the disease and the diagnosis of SARS-CoV-2 infection was 0–2 days for eight of the patients, who were hospitalized on the same day as the diagnosis. A single newborn who was initially asymptomatic tested positive for COVID-19 on her first day of life, likely due to the mother having the infection prior to delivery. At six days old, the infant developed clinical symptoms that were suggestive of COVID-19 and was subsequently admitted to the Pediatric Infectious Diseases ward. Birth occurred at term in all cases, naturally in six cases and by cesarean section in three cases. Two children had a history of acute illnesses, one with measles, and another with an episode of acute enterocolitis. All the patients were admitted to the pediatric ward (Table 1).

The clinical manifestations at admission were fever (8/9 cases) and, less often, cough (5/9 cases), nasal obstruction and rhinorrhea (5/9 cases), and diarrheal stools (2/9 cases). Dyspnea was rare (2/9 cases) and associated with acute bronchiolitis; in one case, it required the administration of oxygen through a facial mask.

Co-infections were identified in three of the patients: one case with *S. pneumoniae* and *H. influenzae* (identified by means of an RT-PCR from respiratory secretions), one case with respiratory syncytial virus (identified by means of a rapid antigen test from nasal secretions), and one case with rotavirus (identified by means of a rapid stool test) in a patient who also had diarrheal stools (Table 2).

Imaging evaluations were performed in six patients, with a normal appearance found in four cases, and pulmonary interstitial accentuation in two.

The laboratory data and the therapeutic and evolutive characteristics of the newborns admitted with COVID-19 are presented in Table 3 and Table 4.

## 4. Discussion

COVID-19 in children is associated with less severe forms of the disease and fewer complications compared to adult patients [1,2,4]. These findings can be explained by several factors, such as children’s decreased expression of the ACE2 receptor, the primary target of the SARS-CoV-2 virus; differences in the levels of transmembrane protease serine 2 (TMPRSS2) between children and adults; and the larger number of other viruses residing in the lung and airway mucosa of children, which could limit the replication of SARS-CoV-2 by means of direct virus-to-virus competition [10]. In addition, children may have stronger innate immune responses and fewer comorbidities, such as smoking-related dysfunctions and obesity [11,12,13]. There are studies that have shown that in the epithelial cells of their upper respiratory tract, children exhibit a higher basal expression of some cellular sensor proteins, such as MDA5, which, upon binding to viral RNA, activate a stronger early innate antiviral response to infection with SARS-CoV-2 than in adults [14,15]. Finally, fetal hemoglobin is speculated to have a role in protecting against coronavirus infection in neonates [16]. Infants with COVID-19 develop mild gastrointestinal or respiratory infections. Pneumonia is not a rare condition, however, and some patients require oxygen therapy. Infant appetite disorders may have a significant impact and be responsible for hospitalization [17,18]. Infants with eating disorders should thus be considered for COVID-19 testing; this could also help control disease spread, as infants may be involved in the transmission of SARS-CoV-2 infection in households [19,20]. The clinical picture of COVID-19 in infants changed significantly between the first two waves of the pandemic [17]. There has been little experience with COVID-19 in young children and neonates. Significant data on COVID-19 in neonates and young children became available during the fifth wave of the pandemic, which was largely caused by the Omicron variant and was more transmissible [21]. Shaiba et al. [22] performed a retrospective study of children younger than 90 days who tested positive for SARS-CoV-2 between March 1 and August 2020 in three hospitals in Saudi Arabia. A total of 36 children were identified; most of them were mildly symptomatic, with fever being the most common presenting feature, similar to what was observed in older children [1,2,5]. Only four (11%) patients had a severe infection, corresponding to a slightly increased incidence compared to that of 6% in previous reports [1,6]. Of these four cases, two were considered to be affected by multisystem inflammatory syndrome in children (MIS-C). Only one death was recorded in this group. The authors argued that the severe course and need for critical care or respiratory support in some of these patients might be explained by the presence of MIS-C, possibly diagnosed in two patients in the post-neonatal age group, and of other conditions, such as cyanotic heart disease and bacterial co-infections. Khoury et al. [23] reported on their experience with 52 COVID-19 patients who were younger than 6 months and admitted during the fifth wave of the COVID-19 pandemic (Omicron variant infection). Of these, 10 patients were younger than 1 month, 33 patients were aged 2–3 months, and 9 patients were aged 3-6 months. The mean age was 8.6 weeks (range of 2–20 weeks). The disease was mild in the neonates and young children, and admission was only required for observation. No patients required ventilation or O_2_ support, their laboratory results were within normal ranges, and there were no complications, including no documented bacterial infections. There were no 30-day readmission cases. They concluded that further studies are needed to reach more definitive conclusions on the severity of COVID-19 in young children, especially regarding the need for full sepsis workups and antibiotic treatments in neonates who are less than 28 days old. Similar results were published by Chen et al. [21]. Panetta et al. [7] reported on 27 infants with COVID-19 who were younger than 1 year, of whom 14 were below 3 months of age, and 13 were 3–12 months of age. The report showed that most of the infants below the age of 3 months presented with mild symptoms (86%), and only eight of them were hospitalized. Most of these infants presented with gastrointestinal symptoms; none of the infants required ICU admission, and only 10 were hospitalized.

Our study evaluated different clinical, biological, therapeutic, and evolutionary aspects of the neonates who were hospitalized for COVID-19. In the epidemiological context of COVID-19, most patients with a SARS-CoV-2 infection are adults, and a low proportion of pediatric patients have been treated; thus, less experience in the management of this disease in neonates has been gained. All nine newborns, four girls and five boys, included this study were born at term, naturally or by cesarean section, with normal birth weights in eight cases and a low birth weight in one, according to the WHO and CDC specifications [24]. Testing for SARS-CoV-2 infection was performed in eight of the patients due to the presence of symptoms that were suggestive of an acute infection; the remaining newborn was tested immediately after birth via cesarean section in the absence of any symptoms, because her mother had COVID-19 before giving birth. Testing in the epidemiological context of the persistence of a SARS-CoV-2 infection and infectious contact enables the isolation of asymptomatic cases of the disease, which are possible sources of infection for susceptible people in the vicinity of a patient, and can trigger cases of healthcare-associated infections, an aspect that is also mentioned in the specialized literature [25]. Later, the newborn presented with suggestive symptoms, a situation that determined her admission to the Infectious Diseases ward.

The clinical onset of the disease occurred at a variable interval after birth, between 5 and 27 days. In most of the patients, their infection with the SARS-CoV-2 virus most likely occurred through direct contact with a source of infection in their immediate neighborhood. An exception was the newborn who tested positive immediately after birth while being asymptomatic, because her mother had a confirmed infection. It is important to mention that if a mother is SARS-CoV-2-positive, her infant should be immediately tested after birth and possibly admitted to the hospital on the same day. The mothers of the children were also hospitalized with their children, and seven mothers tested positive for SARS-CoV-2 infection. The patients were admitted to the Pediatric Infectious Diseases Department, as they did not require intensive care interventions. Regarding the pathological history of the newborns, one of them, aged 22 days, had recently had measles, and another one, aged 26 days, had experienced an episode of acute enterocolitis.

The clinical manifestations at admission were dominated by fever, cough, nasal obstruction, and symptomatology that were suggestive of an acute respiratory tract infection of an unspecified etiology, including SARS-CoV-2 infection. This situation once again emphasizes the need for testing for COVID-19 to achieve an early correct diagnosis for the appropriate interventions. Two children presented with dyspnea at admission, with one of them also requiring oxygen administration at admission. Another two children presented with diarrheal stools as part of the clinical picture of febrile respiratory infection. The diagnosis of co-infections by means of various laboratory tests (RT-PCR of respiratory secretions, respiratory syncytial virus rapid antigen test of respiratory secretions, and rapid stool antigen test for rotavirus) allowed for the appropriate therapeutic interventions, rigorous monitoring, and, very importantly, patient isolation in separate rooms to prevent the occurrence of infections associated with medical assistance, which is a well-known issue that has been mentioned in the specialized literature [26,27,28]. Three patients presented with associated acute bronchiolitis. A co-infection with respiratory syncytial virus, which is known to be frequently involved in this condition, was identified in one of the children [29]. Another patient with bronchiolitis was diagnosed with an *S. pneumoniae* and *H. Influenzae* co-infection, which is very rarely involved in the production of bronchiolitis in young children, and the third had no identified co-infection. This last observation may suggest the direct involvement of SARS-CoV-2 in the production of bronchiolitis, although some studies have shown that infection with SARS-CoV-2 rarely produces acute bronchiolitis, whose evolution is not severe. This is also supported by the finding that during the COVID-19 pandemic, there was a marked decrease in bronchiolitis cases [30].

Regarding the radiological changes that may be detected in patients with an acute SARS-CoV-2 infection, Kurian et al. [31] mentioned that the most common radiological changes encountered in pediatric patients with COVID-19 are ground-glass opacities, consolidations, and peribronchial thickening. Of the six patients in our study who underwent lung radiography, changes were identified in only two of them, represented by an accentuation of the pulmonary interstitium, a finding that is suggestive of a viral infection [32]. In accordance with the existing national protocols for the diagnosis and treatment of COVID-19 at the time of the hospitalization of the patients, the clinical forms of COVID-19 were interpreted as mild and medium [8,9].

The hematological changes found in children with COVID-19 show some differences compared to those that appear in adults. The most common aspects in newborns and infants with COVID-19 with hematological changes are leukopenia and lymphocytosis; lymphopenia is rare and mainly present in older hospitalized children. Thrombocytopenia is rare [33]. In our study, all the patients had normal leukocyte counts; lymphopenia was only present in three children, monocytosis in five, and thrombocytosis in four patients. Different scientific studies have identified certain risk factors for the severe evolution of COVID-19 in children, among which are young age; comorbidities; and elevated C-reactive protein, ferritin, and ALT values at hospitalization [34,35]. The patients in our study did not exhibit changes in the biomarkers that would suggest a severe evolution at admission. Thus, eight of the children had normal C-reactive protein and ALT values. The serum ferritin was elevated in five of the eight patients who underwent this investigation, but the course of the disease was favorable. Regarding possible kidney damage during COVID-19, Saygili et al. [36] highlighted the fact that even pediatric patients with mild or moderate forms of COVID-19 may be at risk of acute kidney injury (AKI), indicating that they require clinical monitoring and monitoring of their biomarkers to detect subclinical renal damage. In our study, all the patients presented with normal values of serum creatinine and, clinically, their diuresis was normal.

No patient received an etiological treatment with remdesivir. During the period in which the patients in our study were hospitalized, remdesivir was not approved for the treatment of SARS-CoV-2 infection in newborns (it was approved for use in newborns by the FDA in February 2024 [37]). The patients in our study were treated with corticotherapy (eight of the children), antibiotics (four of the patients), and symptomatic and supportive therapies (all of the patients). It should be noted that even in the absence of an etiological therapy, the evolution of the disease in the newborns hospitalized with COVID-19 was favorable in all cases, including those cases with viral or bacterial co-infections, which could have produced severe complications [38]. No patient required intensive care interventions. The average length of the hospital stay was 5.11 days, with a longer duration for the patients who had associated acute bronchiolitis or enterocolitis with rotavirus.

Limitations: Our study was a single-center, retrospective study, limited to a time interval of 2 years. The size of our study group was small (nine patients), and there was no control group. There was no long-term follow-up, as no child returned after discharge; so we cannot draw any conclusions regarding any possible long-COVID status in these patients.

## 5. Conclusions

Our study contributes to the accumulation of medical experience regarding different aspects of SARS-CoV-2 infection in newborns in the absence of an etiological treatment. Our data support the observation that infection with SARS-CoV-2 in neonates is a relatively benign condition with a good prognosis. We advocate for the need to carry out investigations into the diagnosis of viral or bacterial co-infections, which could worsen the prognosis, require specific therapeutic interventions, and require isolation in order to prevent the occurrence of healthcare-associated infections. Our study has several limitations and establishes a foundation for future studies on a larger sample of term and premature neonates with different comorbidities.

## Figures and Tables

**Table 1:** Epidemiological and clinical characteristics of nine newborns (1–9) admitted with COVID-19.

	1	2	3	4	5	6	7	8	9
**Age at** **hospital admission (days)**	6	9	10	19	22	25	25	26	28
**Gender**	F	M	F	M	M	M	M	F	F
**Age at the clinical onset of** **the disease (days)**	5	9	10	19	21	23	24	26	27
**Age at the moment of diagnosis** —quick antigen test (QAT), RT-PCR* (days)	1	9	10	19	22	25	25	26	28
**Mother positive for COVID-19?**	Yes	No	Yes	Yes	Yes	Yes	Yes	Yes	No
**Type of COVID-19 diagnostic test**	QAT *, RT-PCR *	QAT *	QAT *	QAT *	QAT *	RT-PCR	QAT *	QAT *	RT-PCR *
**Delivery on time (T) or premature (P)**	T	T	T	T	T	T	T	T	T
**Natural delivery (n) or C-section (CS)**	CS	N	N	N	CS	N	CS	N	N
**Weight at delivery (grams)**	3160	3300	3600	3670	2498	3280	2200	3460	2600
**Admission to clinical ward (CW) or ICU**	CW	CW	CW	CW	CW	CW	CW	CW	CW
**Personal pathological history**	-	-	-	-	Measles	-	-	Acute enterocolitis	-

* RT-PCR = real-time PCR; ICU = intensive care unit; QAT = quick antigen test.

**Table 2:** Clinical and radiological characteristics of newborns admitted with COVID-19.

Patient No.	Fever	Nasal Obstruction/Rhinorrhea	Coughing	Dyspnea	Diarrhea	Other Clinical Manifestations	Oxygen at Admission	Co-Infections	Associated Diseases	Chest X-Ray
1	X *	X	X	- *	X	-	-	-	-	Accentuation of the interstitial markings in the right pulmonary basal area
2	X	X	-	-	-	-	-	-	-	Normal
3	X	-	-	-	-	-	-	-	-	Not performed
4	X	-	-	-	-	-	-	-	-	Not performed
5	X	-	X	-	-	-	-	-	Acute conjunctivitis Anemia	Normal
6	X	X	X	X	-	-	X	*S. pneumoniae*, *H. Influenzae* (RT-PCR—pulmonary secretion)	Acute bronchiolitis	Normal
7	X	X	-	-	-	-	-	RSV (quick test)	Acute bronchiolitis	Diffuse and bilateral accentuation of the interstitial markings
8	X	-	X	-	X	-	-	Rotavirus (quick test from stool)	Acute enterocolitis with rotavirus Anemia	Normal
9	-	X	X	X	-	-	-	-	Acute bronchiolitis	Not performed

* “X”—present, “-“—not present.

**Table 3:** Laboratory data for newborns (1–9) admitted with COVID-19.

Parameter/Patient No.	1	2	3	4	5	6	7	8	9
Seric leucocytes at admission	N	N	N	N	N	N	N	N	N
Seric lymphocytes at admission	↓	N	↓	↓	N	N	N	N	N
Monocytes at admission	↑	↑	↑	N	↑	↑	N	N	N
C-reactive protein at admission	N	N	N	N	N	N	N	↑	N
ALT (GPT) at admission	N	N	N	N	N	N	N	↑	N
LDH at admission	N	-	-	N	N	N	N	N	N
Ferritin at admission	↑	-	↑	N	↑	N	N	↑	↑
Seric creatinine at admission	N	N	N	N	N	N	N	N	N
Thrombocytes at admission	N	N	↑	N	↑	↑	↑	-	N

Legend: N—normal; “-“—not performed, “↓”—decreased, “↑”—increased, ALT—alanine aminotransferase; GPT—glutamate pyruvate transaminase; LDH—lactate dehydrogenase.

**Table 4:** Therapeutic and evolutive characteristics of newborns (1–9) admitted with COVID-19.

Parameter/Patient No.	1	2	3	4	5	6	7	8	9
Corticotherapy	Yes	Yes	Yes	Yes	Yes	Yes	Yes	No	Yes
Antibiotics	No	No	No	No	No	Yes	Yes	Yes	Yes
Fever duration after admission (days)	2	1	1	2	3	1	1	2	-
Hospitalization period (days)	6	3	3	3	6	10	4	6	5
Evolution	F Discharge at personal request	F Discharge at personal request	F	F	F	F	F	F	F

Legend: F—favorable.

## Data Availability

The original contributions presented in this study are included in the article. Further inquiries can be directed to the corresponding author.
